# National genomic evaluation of Korean thoroughbreds through indirect racing phenotype

**DOI:** 10.5713/ab.21.0409

**Published:** 2022-01-21

**Authors:** Jinwoo Lee, Donghyun Shin, Heebal Kim

**Affiliations:** 1 Korea Racing Authority (KRA), Gwacheon 13822, Korea; 2 Department of Agricultural Convergence Technology, Jeonbuk National University, Jeonju 54896, Korea; 3 Department of Agricultural Biotechnology and Research Institute of Agriculture and Life Sciences, Seoul National University, Seoul 08826, Korea; 4 eGnome, Inc., Seoul 05836, Korea

**Keywords:** Genomic Evaluation, Prize Money, Race Trait Improvement, Racehorse, Thoroughbred

## Abstract

**Objective:**

Thoroughbred horses have been bred exclusively for racing in England for a long time. Additionally, because horse racing is a global sport, a healthy leisure activity for ordinary citizens, and a high-value business, systematic racehorse breeding at the population level is a requirement for continuous industrial development. Therefore, we established genomic evaluation system (using prize money as horse racing traits) to produce spirited, agile, and strong racing horse population

**Methods:**

We used phenotypic data from 25,061 Thoroughbred horses (all registered individuals in Korea) that competed in races between 1994 and 2019 at the Korea Racing Authority and constructed pedigree structures. We quantified the improvement in racehorse breeding output by year in Korea, and this aided in the establishment of a high-level horse-fill industry.

**Results:**

We found that pedigree-based best linear unbiased prediction method improved the racing performance of the Thoroughbred population with high accuracy, making it possible to construct an excellent Thoroughbred racehorse population in Korea.

**Conclusion:**

This study could be used to develop an efficient breeding program at the population level for Korean Thoroughbred racehorse populations as well as others.

## INTRODUCTION

Thoroughbred horses had been bred exclusively for racing in17th and 18thcentury England, when native mares were crossbred with imported Oriental stallions of Arabian, Barb, and Turkoman breeding [1]. Horse racing, which originated in Europe, gained popularity all over the world during the 18th and 19th centuries, and today races are held in more than 150 countries worldwide. The popularity of Thoroughbred horse racing now drives a huge multi-sectoral industry. According to the 2018 International Federation of Horseracing Authorities (IFHA) report (www.ifhaonline.org/resources), approximately 94,000 foals of Thoroughbreds are registered each year worldwide, out of which approximately 1,400 are registered in Korea. In Korea, the domestic horse racing industry was gradually popularized under the leadership of the Korea Racing Authority (KRA) and grew into a large industry scaling billions of dollars with total prize money of approximately 188 million dollars, and average prize money per race was close to 100,000 dollars in 2018, according to the IFHA report. It is also recognized as a healthy leisure activity for ordinary citizens and an important resource for the Korean Government. In Korea, the domestic horse racing industry have been trying to secure global competitiveness in terms of quality and scale. In order to achieve this goal, it was important to build and maintain an excellent racehorse population. Therefore, the scientific goal of the Korea Racing Authority is to establish breeding system that can produce racehorses, which can compete internationally with excellent racing ability [2]. In addition, a mid- to long-term plan at the national level in Korea has been established for the production and cultivation of excellent racehorses in accordance with the law.

In this study, based on previous research that race ability is inherited, we tried to build genomic evaluation system that could select excellent individuals and utilizes them as genetic resources for the next generation [3]. Traits related to horse racing performance include finishing time, earning prize money, and rank [4]. And these traits have been well studied in several previous studies [5–9]. Horse racing performance traits are affected by the diverse environmental or economic status of each racing competition, such as racing distance, prize money, inflation rates, grade of the racing group [10,11]. In the case of other livestock, many studies have been conducted on best linear unbiased prediction (BLUP) [12–14], but on racehorses, studies are limited due to perceived difficulty in generation, quantification, and analysis of data. However, a previous study determined a model by combining explanatory variables that affect the racing performance to assess the potential of development of Thoroughbred racehorse breeding based on KRA horse race data [2,15,16]. This study constructed a simple animal model of a combined performance index with these normalized variables for Korean racehorse breeding.

In this study, we analyzed the genetic capacity of Thor oughbred racehorses and quantified the improvement in racehorse breeding output by year in Korea, which was the basis for a high-level horseracing industry and continues to be crucial for further development. To establish breeding system that can produce racehorses, which can compete internationally with excellent racing ability, we applied BLUP, which is a reliable model of the best fit to hundreds of thousands of observed race records. In this genomic evaluation, we used the adjusted prize money as a phenotype instead of the race records to measure the racing ability of each Thoroughbred. Our results indicated that this pedigree-based BLUP method improved racing performance in Thoroughbred populations with high accuracy, and the breeding system based on this evaluation method facilitated the selection and production of excellent stallions and racehorses in Korea. Additionally, this racehorse study contained diverse information related to Thoroughbred racehorses breeding at the population level (KRA Thoroughbred phenotype information, racehorse breeding system information, Thoroughbred sire selection breeding effect, Thoroughbred racehorse BLUP performance).

## MATERIALS AND METHODS

### Thoroughbred racehorse phenotypic data

All racehorses registered under the KRA are accommodated and trained within the confines of KRA racing facilities. KRA is a quasi-market-type public corporation established for promoting horse racing and the development of horse livestock through efficient implementation of plans and fair distribution of horse racing resources. All animals were cared for according to the appropriate animal health and welfare guidelines approved by the Animal Care and Use Committee of the KRA, Korea. The phenotypic data of 25,061 Thoroughbred racehorses (430,106 records) born between 1994 and 2019 was sourced from the KRA and employed in this study. This Thoroughbred racehorse population was composed mostly of individuals aged 2 years and older, with an average inbreeding coefficient of 0.02. Pedigree structures and inbreeding coefficients were obtained using BLUPF90 software [17]. The total number of animals in the pedigree was 39,188 from 3,363stallions and 12,587mares.

**Figure 1:**
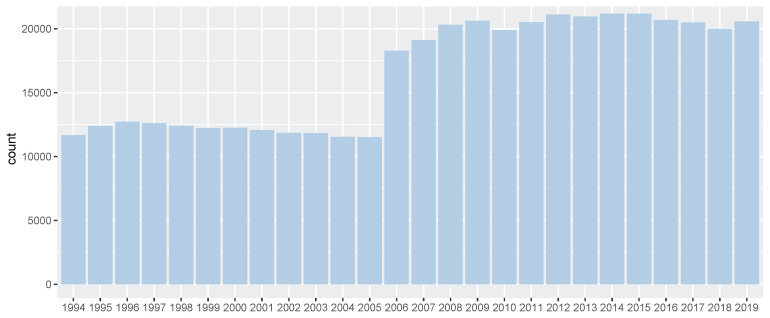
Number of Korea Racing Authority (KRA) horse racing records per year (1994 through 2019). It showed number of race records of all Thoroughbreds in between 1994 and 2019. And since 2006, the number of race records has increased significantly, as one more racetrack has been added.

**Figure 2:**
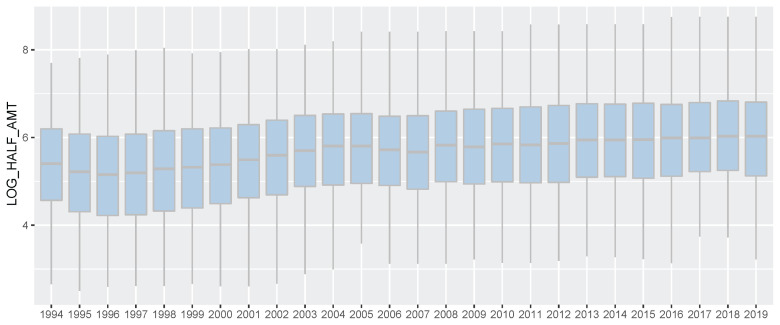
Korea Racing Authority (KRA) horse racing prize index boxplot by year (1994 through 2019). In this study, only the first prize money was the actual prize money and the prize money for each other rank was half of the preceding rank instead of the actual prize money. And then the prize money was log-transformed to construct a normal distribution for best linear unbiased prediction analysis. We regarded this converted prize money as prize index in this horse genomic evaluation for racing ability. And we showed racing prize index for all horses participating in the race in each year.

All the data used belonged to domestic races that took place on dirt tracks. There was diversity in the country of origin of the horses. Racing time, finishing rank, and prizes earned were recorded for all the racing [2]. Before genomic evaluation, we converted racing prize to prize index. In a Korean horse race, usually, 5 to 14 horses participate, and the number of participating horses per race varies depending on the multiple factors. However, regardless of the total number of racehorses, the prize money was paid only for 1st to 5th places. Consequentially, the 6th place and lower horses always received the same race records, but we thought that there was a clear difference in racing ability among the 6th place and lower horses, too. So, in this study, only the first prize money was the actual prize money and the prize money for each other rank was half of the preceding rank instead of the actual prize money. (For example, 2nd place was half of the 1st prize money and 3rd place was half of 2nd place). And then the prize money was log-transformed to construct a normal distribution for BLUP analysis. We regarded this converted prize money as prize index in this horse genomic evaluation for racing ability. The Thoroughbred racehorse records of races between 1994 and 2019 are summarized in Figure 1–2; Supplementary Figure S1–S3.

### Pedigree based best linear unbiased prediction

We performed traditional BLUP analysis with a pedigree-based relationship matrix to estimate breeding values using the entire race record data. The animals were then split into training and validation datasets according to the race start year, and phenotypes in the validation dataset were used to compute the accuracy of the predictions. For traditional pedigree-based genomic evaluations, the following single-trait and repeated-record animal models were fit:


(1) Y=Xb+Za+Jc1+PEc2+e

where Y is a vector of observations (log-transformed prize money index); X is the vector of fixed effects (racecourse, racing year, racing distance, sex, age, gate number, production place, burden weight assignment method, and the number of entries); Z is the vector of random effects, which is an additive genetic effect following a normal distribution $\text{N}(0,\text{A}\sigma_{a}^{2})$. $\sigma_{a}^{2}$ is the additive genetic variance, A is the pedigree-based relationship matrix of individuals, and J and PE are the vectors of the jockey and permanent effect. B, a, c1, and c2 are index matrices associated with X, Z, J, and PE, respectively. E is the vector of the random residual effect that follows a normal distribution $\text{N}(0,\text{I}\sigma_{e}^{2})$. $\sigma_{e}^{2}$ is the residual variance, and I is an identity matrix. This single-trait animal model was used to estimate the breeding value of thoroughbred racehorses for racing prize index.

### Validation of genomic evaluation of Thoroughbred racing performance

We evaluated the predictive ability of the BLUP for Thoroughbred racehorse performance using validation tests. For these tests, we split the racehorse records into training and validation datasets and created five validation datasets. Each validation dataset predicted the Thoroughbred racing performance for 2014 through 2019, respectively. The validation data included animals that started racing after the cut-off date, while the training dataset was composed of animals that started racing before the cut-off date. For example, in the validation dataset for 2014, the validation set included animals that started racing between January 1, 2015, and December 31, 2015, while the training set was composed of animals that started racing before January 1, 2015.When we performed genomic evaluation using the training dataset, we assumed that the phenotypes of animals in the validation set were unknown and only pedigree information was available. Based on the BLUP result, the accuracy of genomic prediction was evaluated by the correlation of the estimated breeding value (EBV) from the validation and the corresponding phenotypes (average of a racing prize index) divided by the square root of the heritability of the trait.


(2) $$\text{Accuracy}=\frac{cor(EBV,Y)}{\sqrt{h^{2}}}$$

## RESULTS

### Phenotypic data and estimation of genetic parameters

Before the genomic evaluation of Thoroughbred racehorses, we estimated the genetic parameters for the racing prize money index of Thoroughbred, the jockey effect, the permanent environmental effect, and the genetic effect, and the errors found were 0.02, 0.17, 0.14, and 0.67 (estimated heritability and repeatability for racing prize money index were 0.14 and 0.31, respectively). Although the racing prize money trait’s heritability was small, this trait was an important phenotype for predicting Thoroughbred racing ability because the repeatability was 0.3118. Based on these parameters, the breeding value of Thoroughbred racehorses was estimated.

### Thoroughbred racehorse sire analysis

**Figure 3:**
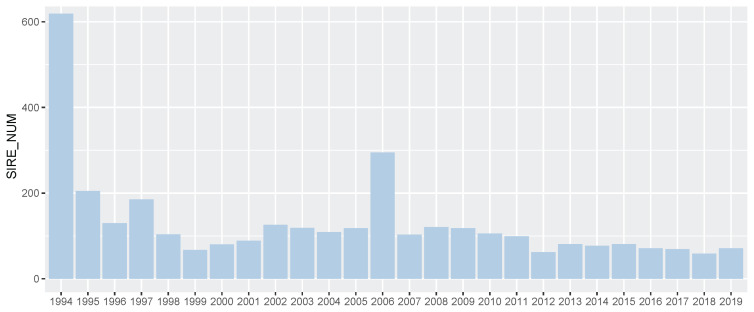
Number of sire per year in Korea Racing Authority (KRA) horse breeding system. We showed the number of sires in the KRA breeding system every year. We considered all stallions to be the stallions of each year based on the year the first progeny started racing.

In 25,061 Thoroughbred racehorses in Korea (from 1994 to 2019), there were 3,364 Thoroughbred sires and approximately 130 sires per year based on the racing start year of the progeny (Figure 3). More Thoroughbred sires were introduced into the Korean population in 1994 (619 sires) and 2006 (295 sires) than other years. Because the first systematic racecourse was established in 1994 (Seoul) and an additional racecourse was established in 2006 (Busan), a large number of racehorses were required and population genetic diversity had to be maintained. For racehorse population structure analysis, we calculated the progeny per Thoroughbred sire and found that only a few sires were dominantly used to produce racehorses (Supplementary Figure S4). Of the 3,364 Thoroughbreds, 3,091 thoroughbred sires had less than 10 progenies, and 3,282 stallions had less than 50 progenies. This was a result of artificial selection by the horse owner for breeding and economic justifications, without a biological reason. To ensure the reliability of the EBV, we checked the number of progenies per Thoroughbred sire, as shown in Supplementary Figure S5. A total of 1,247 of 3,364 Thoroughbred sires had more than 50 racing records of offspring, and 70 sires had more than 1,000 racing records of offspring. The correlation between the number of offspring and the number of race records of offspring per sire was 0.984. From the progeny number and progeny race record number per sire, we found that some Thoroughbred sires were widely used for breeding purposes.

**Figure 4:**
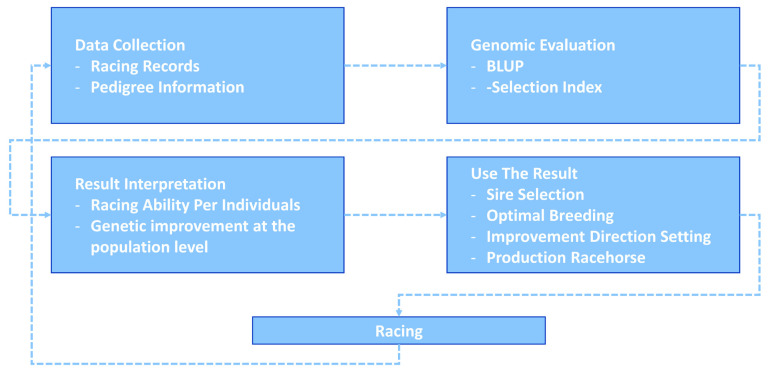
Korea Racing Authority (KRA) racehorse breeding system. This racehorse selection system was actually used to select excellent racehorses, and through this system, data was collected, genetic ability evaluation was performed, and strong breeding centered on male horses was performed.

**Figure 5:**
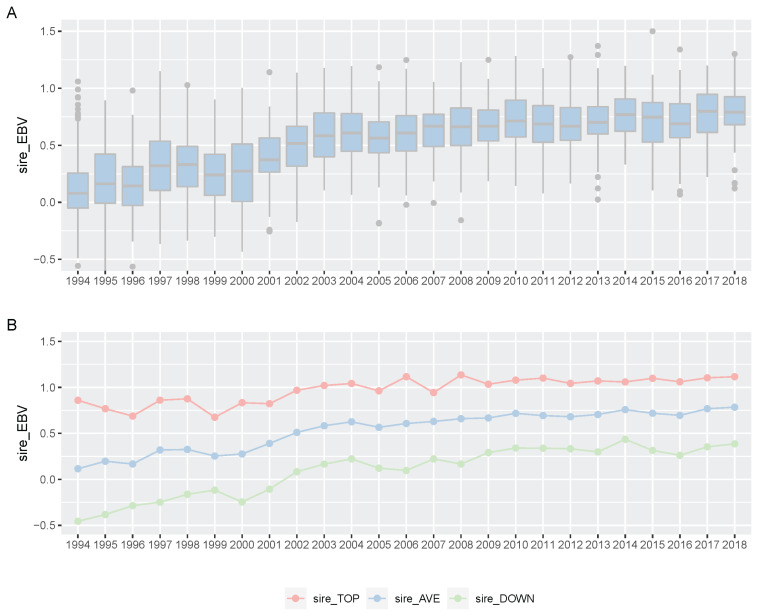
Boxplot of Korea Racing Authority (KRA) sire estimated breeding value (EBV) for racing prize index per year (A) and plot of top, all and down sire EBV mean per year (B). (A) We showed boxplot of all KRA sires EBV per year (1994 through 2019). (B) We indicated EBV average per year of all sires (blue), EBV average of the top 10 sires (red) and average of the bottom 10 sires (green).

**Figure 6:**
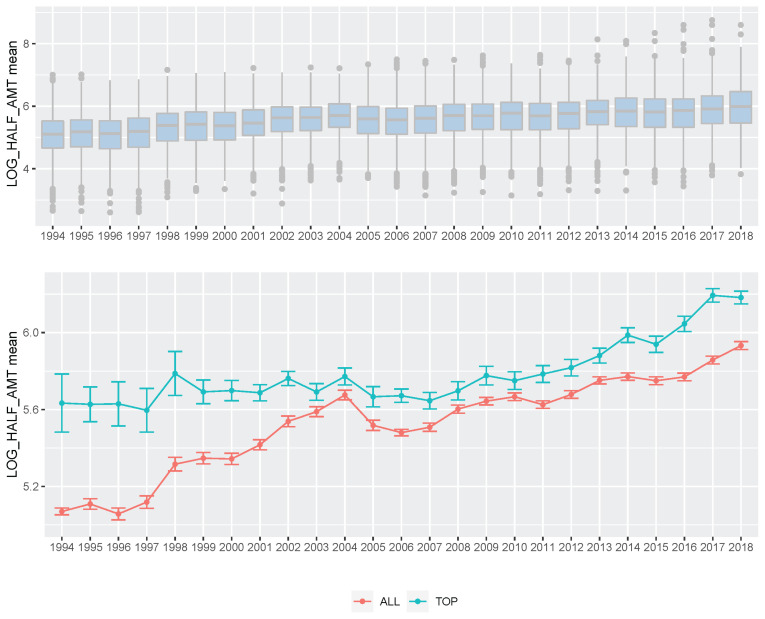
Boxplot of Korea Racing Authority (KRA) racehorse prize index per year (A) and plot of prize index mean per year of KRA racehorse (B). (A) We showed boxplot of all KRA racehorses prize index boxplot per year (1994 through 2018). (B) We indicated average prize index per year of all progeny (red) and progeny of the top sire group (green), respectively.

For 25 years, KRA has applied a breeding program (mainly sire-centered improvement) consisting of selection and mating of Thoroughbred racehorse populations in Korea to improve race performance. A detailed description of the racehorse breeding program is shown in Figure 4. Because of the sire-centered breeding strategy, the racing-related genetic ability of Thoroughbred sires represents the racing ability of the entire racehorse population and is very important in Korean breeding programs. Therefore, KRA estimated sire breeding value based on progeny race record data every year for breeding programs (Figure 5), and it was found that the average sire breeding value for racing prize money index increased from 0.115 in 1994 to 0.769 in 2019. For analysis of the average race-related genetic ability of the Thoroughbred sires, we considered it necessary to observe the top and bottom sire groups to identify the effect of the racehorse breeding program. In the 25 years since the start of the horse race, we observed that the average breeding value for racing prize money index of the top (10 selected sires based on EBV per year) and bottom (10 selected individuals per year) sire groups increased continuously. In the sire EBV analysis, we found that the average breeding value of the bottom sire group (from −0.456 to 0.386) had a higher average rate of increase than of the top sire group (from 0.860 to 1.129) between 1994 and 2019. In addition, we found that the difference in the average breeding value between the top and bottom sire groups gradually decreased from 1994 to 2019. In this sire data analysis, since the deviation of progeny number per sire was very large, the progeny number difference among sires could have affected the sire breeding value estimation. However, there was no correlation between the progeny number per sire and sire breeding values (correlation value = 0.117). Therefore, the number of offspring was not considered in the estimation of sire breeding value in this study. Thoroughbred sires with better racing abilities were selected each year, and a direct correlation of their abilities with the performance of their progenies was determined. To identify the effect of the sire-centered breeding strategy for Thoroughbred racehorse populations in Korea, we analyzed the prize money index of the progeny per year (Figure 6). It was found that the average prize money index (log-transformed, observations in BLUP) gradually increased from 5.070 in1994 to 5.790 in 2019. Then, we separated the progeny of sire in the top group, calculated the average of the racing prize money index, and compared it with the total progeny (Figure 6). The average prize money index of the progeny of the top sire group per year increased from 1994 (average = 5.633) to 2019 (average = 6.057). However, the increase in the progeny of the top sire group (from 5.633 to 5.789) was lower than that of the total progeny (from 5.070 to 5.789) in 25 years. In particular, the average prize money index of the progeny of the top sire group showed little increase from 1994 to 2007 and then increased at a higher rate since 2007. On the other hand, the average prize money index of the progeny of all sires increased rapidly from 1994 to 2004, and the rate of increase slowed down since 2004, as compared to the top sire group. Therefore, in the case of differences between total progeny and top sire group progeny, the largest difference was observed in 1994, and the difference sharply decreased until 2004, and there remained a small difference between 2005 and 2018.

### Thoroughbred racehorse BLUP predictive accuracy

**Figure 7:**
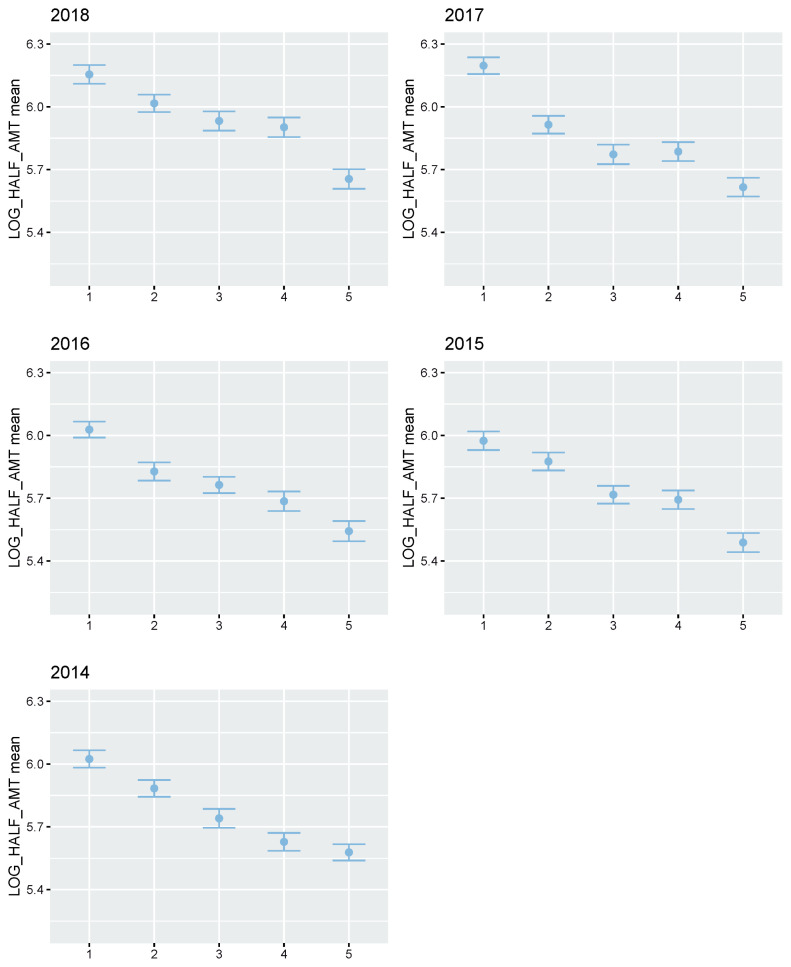
Plot of Korea Racing Authority (KRA) racehorse real prize index mean of each group after grading using estimated breeding value (EBV) for BLUP validation by year (1994 through 2018). In genomic evaluation for validation, estimated EBV was from BLUP estimation without racehorse racing records of test population. This test population was divided into five groups as follows. Grade 1, top 0% to 20%; Grade 2, top 20% to 40%; Grade 3, top 40% to 60%; Grade 4, top 60% to 80%; Grade 5, top 80% to 100%.

We showed the reliability of the prediction by calculating the accuracy of the Thoroughbred racehorse genomic evaluation. The accuracies of genomic evaluation per year (2014 through 2018) for the racing prize money index using pedigree-based BLUP are presented in Supplementary Table S1. The average accuracies of the pedigree-based BLUP results in the last five years were 0.610, 0.799, 0.670, 0.713, and 0.688 in 2014, 2015, 2016, 2017, and 2018, respectively. We split the test dataset into two datasets with the number of racing records of each racehorse (test dataset 1: 10 races or more horses and test dataset 2: less than 10 racehorses). It was noted that the prediction accuracies of test dataset 1 were higher than those of test dataset 2 in 2017 and 2018, while the accuracies of test dataset 2 were higher than test dataset 1 in 2014, 2015, and 2016.Therefore, we found that the number of racehorses in records had no clear correlation with the accuracy of genomic evaluation. Additionally, to test the actual predictive power of genomic evaluation, we divided each validation set into five grades (Grade 1, top 0% to 20%; Grade 2, top 20% to 40%; Grade 3, top 40% to 60%; Grade 4, top 60% to 80%; Grade 5, top 80% to 100%) using the breeding value from the test set and calculated the average of the phenotypes for each grade (Figure 7; Supplementary Table S2). We found that the phenotype of five grades was clearly distinguished by their breeding values in all five years (2014 through 2018). Although the heritability of the racing prize money index from Korean horse race records was not high at 0.143, it was very useful in predicting the actual Thoroughbred racehorse capacity.

### Evaluation of sires selected from retired Korean racehorse population

**Figure 8:**
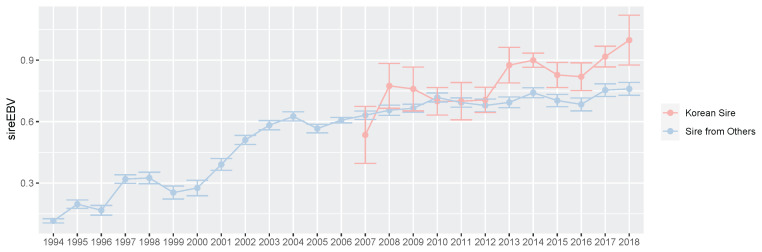
Plot of the average and standard error of imported sire estimated breeding value (EBV) and Korean sire EBV. We showed the average and standard error of EBV for prize index of sires from other countries (2014 through 2018, blue) and Korea Racing Authority (KRA) sires s (2007 through 2018, red) per year. In this study, we defined Korean sire which selected based on racing records in only Korea.

For a long time, most sires of Thoroughbred populations in Korea were selected and used based only on the race records outside Korea or on pedigree information. If the environment of data generation is different from the actual racing environment, the breeding efficiency may be reduced regardless of how well the genetic ability analysis is performed. To overcome this limitation, KRA built a system that produces excellent thoroughbred sires using Korean race records in racehorse breeding programs for over 10 years. In this study, we evaluated sires selected from a retired Korean racehorse population, which had racing experience, based on race records in the Korean racecourse. We found that there were only 70 of 3,364 sires (1994 through 2019) in the Korean Thoroughbred population and their progeny started the race in Korea in 2007. We compared the EBV of these sires to the overall sire EBVs between 2007 and 2018 (Figure 8). From these results, we found that the EBV average of the sire from the Korean population and of the overall sire were similar from 2007 to 2012. However, the EBV average of sires selected in Korea was slightly higher than that of the overall sire after 2013.

## DISCUSSION

In this study, we performed the genomic evaluation of a Korean Thoroughbred population containing sires and racehorses based on race records between 1994 and 2019. For 26 years, there were 25,061 racehorses and 430,106 racing records in KRA. As the number of horse racecourses increased, the number of race records and racehorses increased after 2006 (race records per year: 12,095.08 records in 1994 to 20,513.69 records in 2019; racehorses per year: 624.17 individuals in 1994 to 1,221.77 individuals in 2019). Since the system in all racecourses was the same, we used racecourse information as a constant factor, and data from all racecourse were used in racehorse genomics evaluation. Although several previous studies have reported that using the individual best racing finish time is important for genomic evaluation [18,19], it seemed impractical in Korea because there were no specific racing distances here [20]. Therefore, we used the log-transformed prize money index as a response variable in this genomics evaluation. The estimated heritability of the racing time of Japanese Thoroughbred racehorses was in the range of 0.09 to 0.22 [21] and those of Brazilian Thoroughbred horses were in the range of 0.10 to 0.32 [7]. It was also reported that the heritability of the racing time of Iranian Thoroughbreds was in the range of 0.09 to 0.13 [8]. Given that the traits used in this study, such as racing time, were closely related to racing performance, it is reasonable to conclude that the heritability of the racing prize money index was similar to that of racing time. We calculated the log-transformed prize money index of all racehorses per year in this study (Figure 2), and found that the average prize money index increased gradually from 5.317 in 1994 to 5.967 in 2019. Although the average prize index of all individuals was increasing, we did not think that the average increase was a problem because we adjusted prize index to years when estimating the breeding value. The fact that the average EBV has generally increased every year for 25 years (Figure 5), and that the average prize money of the top sire group’s progeny has steadily increased (Figure 6), is evidence that our horse genomic evaluation is very useful. And the histogram of the log-transformed prize money index showed a normal distribution. Therefore, it was judged that this index related to racing performance was suitable for use as response variable information in genomic evaluation. Another characteristic of race records in this study was that the distribution of the number of records per individual was skewed (highly concentrated between 0 and 10). Due to the features of racehorse management, horses with good results immediately after the start of the race could continue to participate in the race. However, most of them were retired and could no longer participate in the race. Consequentially, there were a small number of horses with many racing records. We checked the distribution of phenotypes for each group with respect to all fixed effects used in the genomic evaluation. We confirmed that there were few outliers in our racing record data of Thoroughbred racehorse population in Korea, so we decided that this large-scale racehorse data was suitable for estimating the breeding value. In this study, genomic evaluation was performed with all horse race record data for academic purposes, and this method was applied to racehorse population management in Korea (Figure 4). Every year, all race records and pedigree information were collected, and genomic evaluation of all Thoroughbred racehorse breeding in Korea was performed. Based on this result, we could estimate the genetic racing ability of each racehorse and sire. The racehorses with excellent genetic ability were selected to raise the racing capacity at the population level. A comprehensive breeding program was established at the population level, and racehorses were brought from domestic farms or outside superior groups, and thousands of races per year were run based on the Thoroughbred racehorse population administration. Such evaluation-based race execution was carried out every year for the past 26 years.

From the genomic evaluation of the Korean Thoroughbred racehorse population, it was found that the average sire breeding value gradually and slightly increased from 1994 to 2019 (Figure 5). This meant that the genetic ability of the Thoroughbred racehorse population in Korea was improved by selecting an excellent sire. This genetic improvement in the racing ability of Korean Thoroughbreds racehorses was consistent with previous studies based on Korean Thoroughbreds racing time records data [20] and that of other populations [7,22,23]. Interestingly, it was found that the difference in the average breeding value between the top and bottom sire groups gradually decreased from 1994 to 2019. Horse racing results should not be predictable because they are closely related to the gambling industry. Therefore, it was important to reduce the difference in genetic racing ability between individuals. In addition, since the overall average also increased, it was judged that the direction of sire-centered breeding was appropriate. From sire number analysis, we could see that the number of races and racehorses increased significantly after 2006, but the number of sires did not increase, except in 2006. This would have led to a stronger selection from an increased number of racehorses after 2006. In this study, the racing performance of the progeny of the top sire group did not increase much before 2006 (only the overall average increased rapidly), but after 2006, their performance steadily increased (Figure 6).

It is crucial to evaluate and predict the racing performance of racehorses before the actual race, as well as to construct a good sire population for breeding programs. Therefore, we intended to show the reliability of the prediction by calculating the accuracy of the Thoroughbred genomics evaluation using five validation datasets. The average accuracy using pedigree-based BLUP in the last five years was 0.699 (Supplementary Table S1). However, because of the low heritability of the racing prize money index, it was thought that the accuracy of prediction would decrease in real-life applications. So, in this study, to test the reliability of prediction using genomic evaluation, we divided each validation set into five grades using the breeding value from the test set and compared the average of phenotypes to the EBV for each grade (Figure 7; Supplementary Table S2). We could see that the five grades categorized by breeding value are clearly distinguished by phenotype in all five years. Although the heritability of racing prize money index for Korean Thoroughbred racehorses was not high, since the variance of the permanent environmental effect was 0.169 and the racehorses in the Korean Thoroughbred population shared a common environment, our EBV could be used to predict and evaluate the racing performance of racehorses.

Originally, Thoroughbred racehorses were bred exclusively for racing in England, and because of the lack of opportunities to build up sufficient know-how and the limitation of group size, many countries around the world that held horse races did not have superior sire or racehorse production and advanced breeding systems. This is because establishing a sire or racehorse production and the breeding system is different from holding horse racing. Therefore, we selected sires from a foreign population to improve the racing performance of the Thoroughbred racehorse population in Korea for approximately 20 years. However, the racing environment is very diverse, and if the sires or racehorses have to be imported, the host has to rely on a race record from a different environment. Since traits for measuring racing performance may differ with the racing environment, breeding systems using imported sires or horses may be less efficient in population management. From a genetic point of view, sire selection is the most important decision by livestock breeder, as the level of a stallion determines the average ability of the next generation. Most of the genetic improvements in domestic animal populations, including horses, have been a direct result of sire selection, so it is very important to maintain an excellent and suitable sire. This is why KRA has been carrying out a strong breeding strategy centered on sires. Although the Korean population was not originally a horse racing population, KRA built a system that could produce sires based on large-scale Korean race record data and was able to select sires with better-than-average breeding value. However, since it was still only a small proportion, it was necessary to continuously select sires from the Korean Thoroughbred racehorse population and analyze the race patterns of their descendants.

In this study, we could have collected enormous horse racing data from 1994 to recently and we thought that such near-perfect race and pedigree data of thoroughbred were very rare in the world. Nevertheless, there had been no systematic genomic evaluation for breeding program in Korea. So, we aimed to establish genomic evaluation system based on large-scale Korean racing records (using the prize money index as a novel phenotype) and performed this method for many years to test whether it was suitable to construct and improve the Thoroughbred racehorse population. Our results indicated that this evaluation method was judged to be very useful in measuring the racing characteristics of horses and suggested the possibility of producing superior sires selected from a retired Korean racehorse population. We hope that this study will be used to design and construct efficient breeding programs at the population level for Korean and other Thoroughbred racehorse populations. Additionally, we plan to study various methods (multi-trait BLUP, genomic BLUP, single-step BLUP) containing single nucleotide polymorphism data or using other phenotype or other genomic evaluation model contacting different effects) using large-scale genome data in the future starting from this study.

## SUPPLEMENTARY MATERIAL

**Supplementary Figure S1**. Histogram of KRA horse racing prize index **Supplementary Figure S2**. Histogram of KRA horse racing record number per individual **Supplementary Figure S3**. KRA horse racing prize index boxplot per covariate **Supplementary Figure S4**. Histogram of progeny number per sire of Korea **Supplementary Figure S5**. Histogram of progeny racing records number per KRA sire **Supplementary Table S1**. Cross-validation results of racehorses of Korea that participated in the first race in recent five years **Supplementary Table S2**. KRA prize index mean of each group after grading using EBV
