# Supplementary material for: National genomic evaluation of Korean thoroughbreds through indirect racing phenotype
Source: Anim Biosci. 2022 Jan 21;35(5):659–69. doi: 10.5713/ab.21.0409 (PMC9065785; doi:10.5713/ab.21.0409)
Supplement: Supplementary file 1 [file ab-21-0409-suppl.pdf]

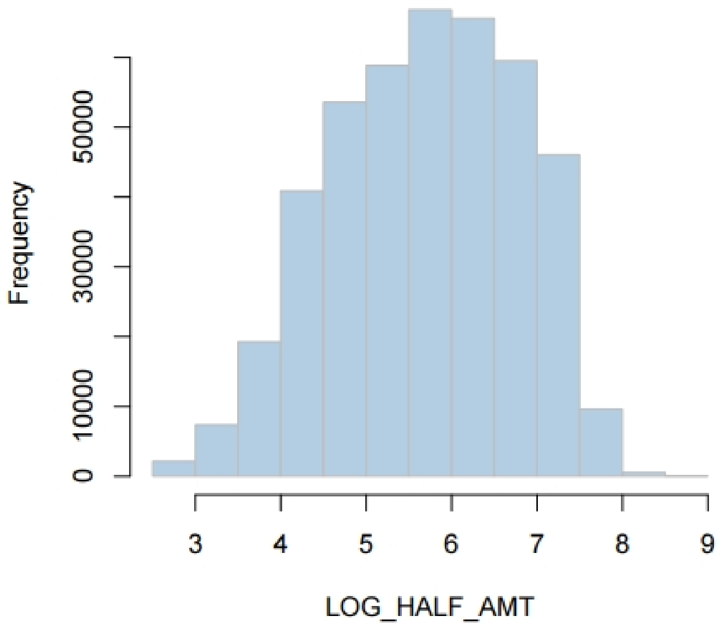


**Supplementary Figure 1**. Histogram of KRA horse racing prize index

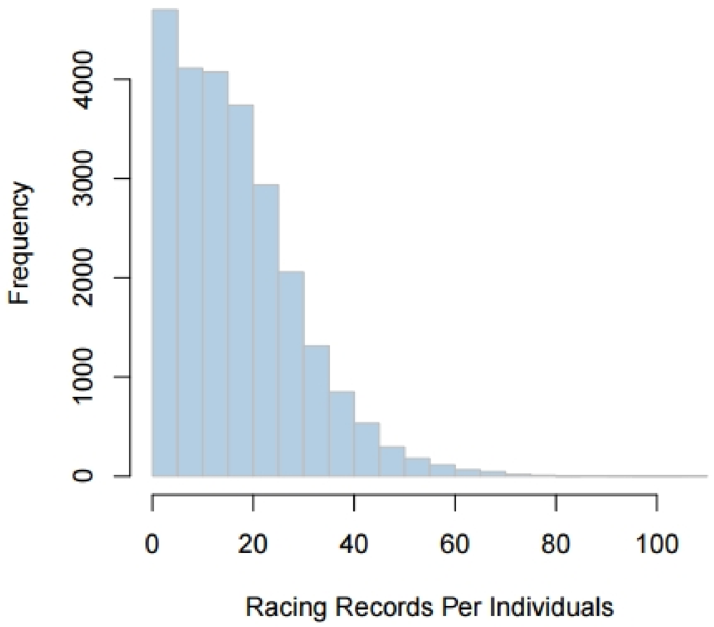


**Supplementary Figure 2**. Histogram of KRA horse racing record number per individual

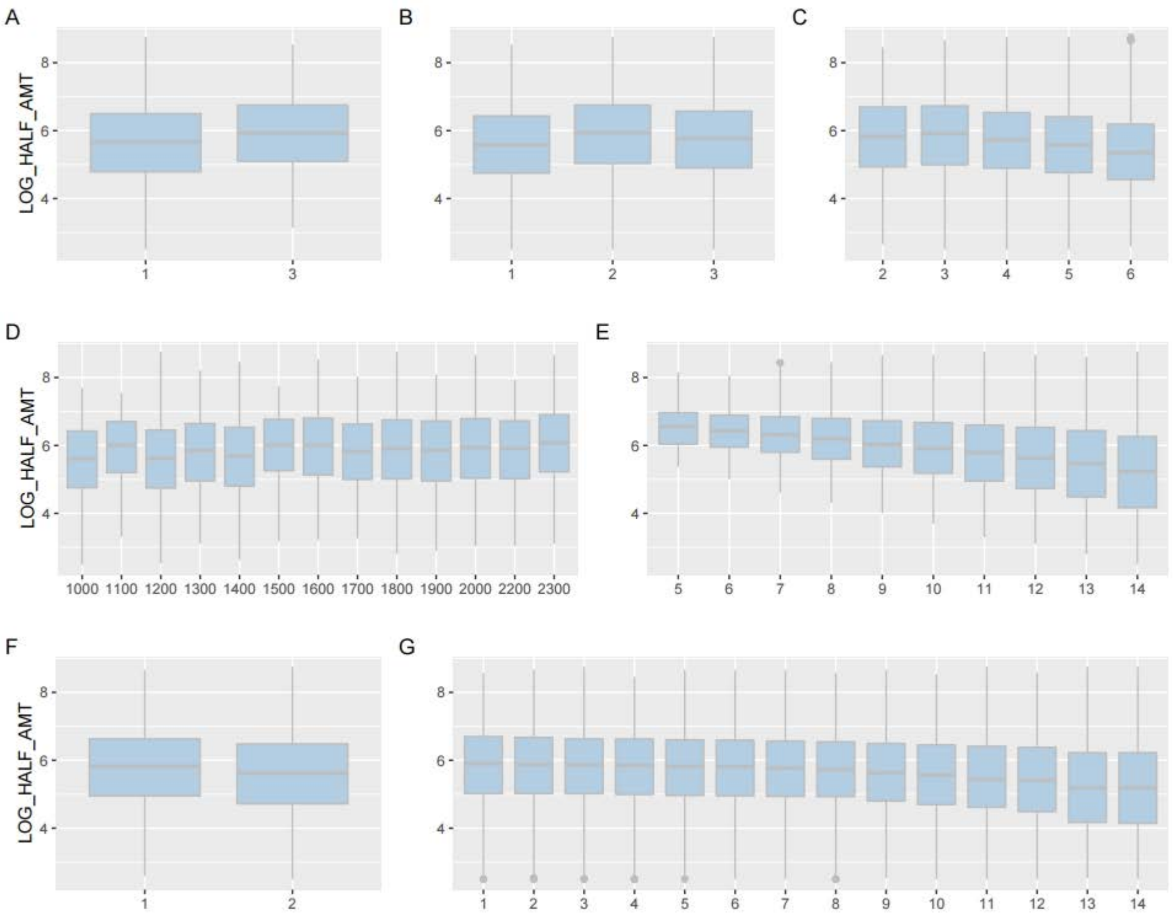


**Supplementary Figure 3**. KRA horse racing prize index boxplot per covariate (A) MEET(Seoul / Busan-Gyeongnam), (B) SEX(female, male, gelding), (C) AGE(2-year-olds, 3-year-olds, 4-year-olds, 5-year-olds, more than 6-year-olds) (D) DISTANCE (E) Number of Runners (F) Classification(domestic-bred / foreign-bred) (G) Gate Number

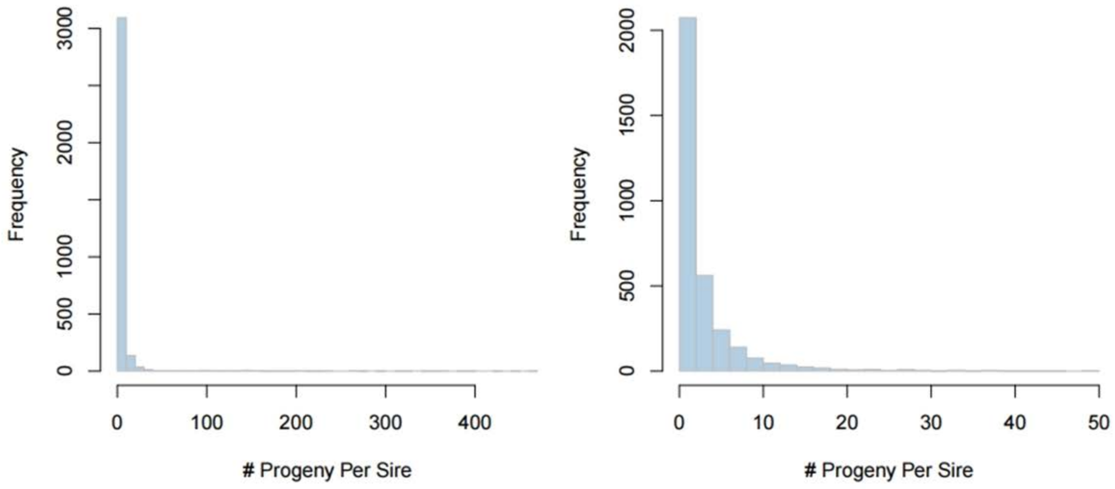


**Supplementary Figure 4**. Histogram of progeny number per sire of Korea (All: 3,364 & 0~50: 3,282)

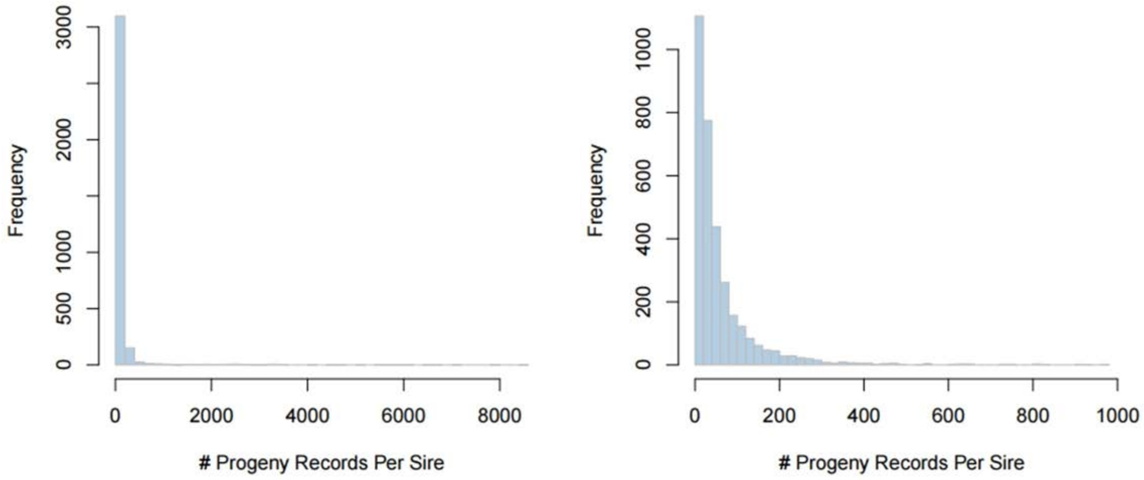


**Supplementary Figure 5**. Histogram of progeny racing records number per KRA sire (All: 3,364 & 0~1000: 3,294)

**Supplementary Table 1**. Cross-validation results of racehorses of Korea that participated in the first race in recent five years

*COR: correlation between EBV (from BLUP estimation without racehorse racing records of the test population) and KRA prize Index mean, **ACC: realized accuracy

| Year | All | | | 10 races or more horse | | | less than 10 races horse | | |
|---|---|---|---|---|---|---|---|---|---|
| | n | COR* | ACC** | N | COR | ACC | n | COR | ACC |
| 2018 | 1,279 | 0.214 | 0.610 | 640 | 0.202 | 0.575 | 639 | 0.242 | 0.690 |
| 2017 | 1,256 | 0.281 | 0.799 | 818 | 0.256 | 0.729 | 438 | 0.274 | 0.780 |
| 2016 | 1,259 | 0.235 | 0.670 | 817 | 0.250 | 0.710 | 442 | 0.157 | 0.446 |
| 2015 | 1,230 | 0.250 | 0.713 | 843 | 0.248 | 0.707 | 387 | 0.219 | 0.625 |
| 2014 | 1,292 | 0.241 | 0.688 | 879 | 0.307 | 0.875 | 416 | 0.133 | 0.379 |

**Supplementary Table 2**. KRA prize index mean of each group after grading using EBV (from BLUP estimation without racehorse racing records of test population). Grade 1: top 0~20%, Grade 2: top 20~40%, Grade 3: top 40~60%, Grade 4: top 60~80%, Grade 5: top 80~100%

| 2018 | | | | |
|---|---|---|---|---|
| Grade | n | MEAN | sd | se |
| 1 | 256 | 6.155 | 0.713 | 0.045 |
| 2 | 256 | 6.016 | 0.667 | 0.042 |
| 3 | 256 | 5.933 | 0.735 | 0.046 |
| 4 | 256 | 5.902 | 0.743 | 0.046 |
| 5 | 255 | 5.655 | 0.748 | 0.047 |
| 2017 | | | | |
| Grade | n | MEAN | sd | se |
| 1 | 252 | 6.197 | 0.636 | 0.04 |
| 2 | 251 | 5.914 | 0.669 | 0.042 |
| 3 | 251 | 5.773 | 0.739 | 0.047 |
| 4 | 251 | 5.786 | 0.711 | 0.045 |
| 5 | 251 | 5.616 | 0.707 | 0.045 |
| 2016 | | | | |
| Grade | n | MEAN | sd | se |
| 1 | 252 | 6.028 | 0.602 | 0.038 |
| 2 | 252 | 5.828 | 0.689 | 0.043 |
| 3 | 252 | 5.764 | 0.617 | 0.039 |
| 4 | 252 | 5.686 | 0.737 | 0.046 |
| 5 | 251 | 5.543 | 0.762 | 0.048 |
| 2015 | | | | |
| Grade | n | MEAN | sd | se |
| 1 | 246 | 5.974 | 0.695 | 0.044 |

| | | | | |
|---|---|---|---|---|
| 2 | 246 | 5.876 | 0.668 | 0.043 |
| 3 | 246 | 5.717 | 0.667 | 0.043 |
| 4 | 246 | 5.693 | 0.697 | 0.044 |
| 5 | 246 | 5.488 | 0.715 | 0.046 |
| 2014 | | | | |
| Grade | n | MEAN | sd | se |
| 1 | 259 | 6.024 | 0.667 | 0.041 |
| 2 | 259 | 5.884 | 0.643 | 0.04 |
| 3 | 258 | 5.741 | 0.724 | 0.045 |
| 4 | 258 | 5.628 | 0.684 | 0.043 |
| 5 | 258 | 5.578 | 0.624 | 0.039 |
